# Editorial: EBV-induced T cell immunity in cancers

**DOI:** 10.3389/fimmu.2024.1454792

**Published:** 2024-07-09

**Authors:** Baochun Zhang, Josef Mautner, Christian Münz

**Affiliations:** ^1^ Department of Medical Oncology and Department of Cancer Immunology and Virology, Dana-Farber Cancer Institute, Harvard Medical School, Boston, MA, United States; ^2^ Institute of Virology, Helmholtz Munich, Munich, Germany; ^3^ Viral Immunobiology, Institute of Experimental Immunology, University of Zürich, Zürich, Switzerland

**Keywords:** Epstein-Barr virus (EBV), T cell, EBV-specific, tumor-associated antigens (TAA), TAA-specific, patient-derived xenograft (PDX), antibody-epitope conjugate (AEC)

The intricate interplay between viral infections and cancer development has long been a subject of intense research. As the first identified and thus longest studied human tumor virus, Epstein-Barr virus (EBV) has been etiologically linked to multiple malignancies of lymphoid and epithelial origins (1, 2). Yet, this virus is distinct from other oncogenic viruses and continues to offer surprises, particularly in the breadth and strength of immune response it induces. The recent collection of six papers on the Research Topic “EBV-Induced T Cell Immunity in Cancers” provides an update and overview of the current understanding and novel insights into EBV-elicited immune responses, with a particular focus on T cell-mediated immunity and its implications for cancer immune surveillance and therapy.

In this Research Topic, Zhang and Xu provide a comprehensive overview of EBV-elicited T cell responses, including their specificities to the viral antigens with relevance to EBV-associated malignancies of different latency states and their therapeutic use in treating these cancers, well established in the literature. They also discuss recent findings in the field (3–5) indicating that EBV may induce T cell responses to a wide range of tumor-associated antigens (TAAs), a set of cellular antigens often shared by multiple cancers. Further included in the discussion are implications of the virus specific and TAA specific T cells in prevention and therapy of EBV-related as well as EBV-unrelated cancers. These underscore the complexity of the immune response to EBV and broaden its significance in cancer progression and management, even in cancers not directly caused by the virus.

In recent literature, it has been well noted that attempts to generate patient-derived xenograft (PDX) lines by transplanting EBV-unrelated tumors into immunodeficient mice often (in ~30% of cases) led to the outgrowth of EBV-transformed B cells (reflecting infiltration of EBV-carrying B cells in the original tumor biopsy). In one such case, Aran et al. conduct in-depth analysis of the T cell responses, demonstrating that the initial tumor-infiltrating T cells also infiltrated and clonally expanded in the EBV^+^ B-cell tumor, and some of them seemed to target certain shared TAAs. These PDX models may provide an avenue to study EBV-induced TAA-specific T cells in human cancers not classically associated with the virus.

EBV infects ~95% of humans by adulthood. After clearing acute infection, EBV-specific T cells form long-term memory and constantly surveil against viral reactivation throughout the rest of life. An innovative strategy is being explored to redirect these virus-specific T cells to target cancers not associated with the virus. This strategy uses specially designed antibody-epitope conjugates (AECs), where immunodominant viral epitopes are conjugated to tumor-targeting antibodies. After binding of the AECs to the antibody target on tumor cells, the viral epitope peptides are proteolytically released, and presented by MHCs, allowing tumor cells to be targeted by the virus-specific T cells. To facilitate AEC development, van der Wulp et al. test three methods of AEC generation, including chemical conjugation via maleimide reaction, enzymatic conjugation using sortase A, and genetic fusion. The stability, specificity, efficiency, and limitation of the different conjugation approaches are compared and discussed, providing valuable insights for further research and development in this area.

Two additional publications discuss adoptive EBV-specific T cell transfer to treat virus associated lymphoproliferations after hematopoietic stem cell (HCT) or solid organ transplantation (SOT). In HCT recipients, the adoptive transfer of EBV-specific T cells from the HCT donor has been shown to induce durable remissions of EBV^+^ lymphomas, even in patients with Rituximab-refractory disease. However, immunotherapy with transplant donor-derived T cells has several limitations. The process of isolating/generating EBV-specific T cells may be too lengthy for the often rapidly progressing EBV^+^ lymphomas or may be impossible if the HCT donor is EBV-negative or a cord blood allograft. More recently, EBV-specific T cells generated from allogeneic 3^rd^ party donors have been adoptively transferred and demonstrated similar clinical efficacy. In the review by O’Reilly et al., latest results from clinical trials of 3^rd^ party and donor-derived EBV-specific T cells are presented. The authors compare attributes and limitations of each product in terms of access, safety, response rate, and durability, and discuss potential donor and host factors contributing to T cell persistence. Lastly, factors contributing to treatment failures and approaches to prevent or salvage relapse are examined, and strategies to further improve virus-specific immunotherapies are outlined.

An allogeneic HCT donor derived T cell product and its effects in HCT recipients is described in the study of Gerbitz et al. In their randomized phase I/IIa MULTIVIR-01 study 33 patients that had received allogeneic HCTs received T cells that had been expanded with EBV and cytomegalovirus (CMV) derived peptides, in order to prevent disease manifestations of both virus reactivations. No severe side effects of the treatment were reported. However, due to the limited patient number, mainly resulting from low recruitment in the face of efficient CMV prophylaxis alternatives, no clear effects of prophylactic T cell transfer on EBV and CMV reactivation could be observed. Nevertheless, the trial has generated EBV and CMV specific HCT donor T cell lines that can now even be explored as virus specific third-party T cell products.

In the final publication of this Research Topic Kong and Guilino-Roth discuss the different EBV latency patterns and T cell responses that can target them. Furthermore, strategies to induce additional viral antigen expression in EBV latency I tumor such as Burkitt’s lymphoma, either from other latent genes or after lytic reactivation, are reviewed.

Collectively, these six papers underscore the importance of EBV-induced T cell immunity in the context of cancer. They highlight the potential for EBV-specific T cell responses to be harnessed for therapeutic purposes, whether through direct targeting of EBV-associated malignancies or by mitigating the complications of viral reactivation in immunocompromised patients, or even redirecting their activity to EBV-unrelated cancers. The research also points to the need for a deeper understanding of the potential role of EBV-induced TAA-specific T cells in cancers not traditionally linked to the virus, suggesting that its influence may extend beyond the well-known EBV-associated malignancies.

In conclusion, this Research Topic represents a critical step forward in the field of oncology and immunology. The findings from these papers provide a foundation for future research and clinical applications, with the ultimate goal of harnessing the power of the immune system to combat cancer more effectively. As we move forward, it is imperative that the scientific community continues to build upon this knowledge, exploring the full potential of EBV-induced T cell immunity in the fight against cancer.

## Author contributions

BZ: Writing – original draft, Writing – review & editing. JM: Writing – original draft, Writing – review & editing. CM: Writing – original draft, Writing – review & editing.
